# Supplementary material for: Transcriptomic Insights into Mechanisms of Early Seed Maturation in the Garden Pea (Pisum sativum L.)
Source: Cells. 2020 Mar 23;9(3):779. doi: 10.3390/cells9030779 (PMC7140803; doi:10.3390/cells9030779)

Zhewan−1,25(1)

Global Summary

%DE = 0.23

genes with fdr < 0.2 = 487 (227 + / 260 −)

genes with fdr < 0.1 = 424 (203 + / 221 −)

genes with fdr < 0.05 = 406 (191 + / 215 −)

genes with fdr < 0.01 = 351 (173 + / 178 −)

genes in genesets = 0

<FC> = 0

<t−score> = 0

<p−value> = 0.01

<fdr> = 0.77

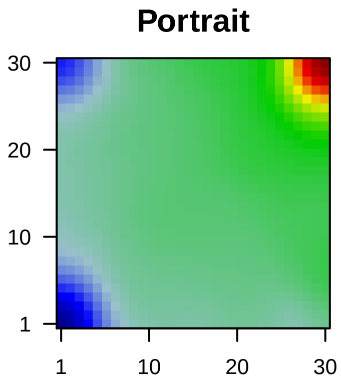

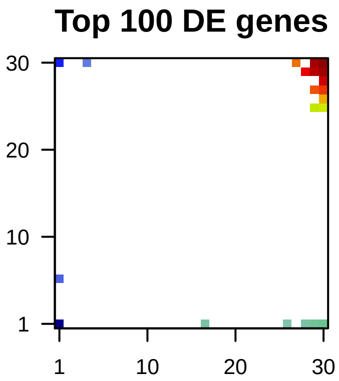

Global Genelist

Rank	ID	log(FC)	p−value	fdr	Metagene	Description
1	TRINITY_DN2	−0.53	2e−16	3e−15	1 x 1	
2	TRINITY_DN1	−0.72	2e−16	3e−15	1 x 1	
3	TRINITY_DN9	0.69	2e−16	3e−15	30 x 30	
4	TRINITY_DN2	1.01	2e−16	3e−15	30 x 30	
5	TRINITY_DN7	−0.98	2e−16	3e−15	30 x 1	
6	TRINITY_DN7	2.62	2e−16	3e−15	30 x 30	
7	TRINITY_DN5	3.91	2e−16	3e−15	30 x 30	
8	TRINITY_DN5	−1.63	2e−16	3e−15	1 x 1	
9	TRINITY_DN5	−0.71	2e−16	3e−15	1 x 30	
10	TRINITY_DN7	1.12	2e−16	3e−15	30 x 30	
11	TRINITY_DN7	−1.28	2e−16	3e−15	1 x 1	
12	TRINITY_DN4	−4.28	2e−16	3e−15	1 x 1	
13	TRINITY_DN9	−0.8	2e−16	3e−15	1 x 1	
14	TRINITY_DN9	0.95	2e−16	3e−15	30 x 30	
15	TRINITY_DN9	1.83	2e−16	3e−15	30 x 30	
16	TRINITY_DN9	0.52	2e−16	3e−15	27 x 30	
17	TRINITY_DN2	0.52	2e−16	3e−15	29 x 30	
18	TRINITY_DN2	−0.73	2e−16	3e−15	1 x 1	
19	TRINITY_DN2	0.71	2e−16	3e−15	30 x 1	
20	TRINITY_DN2	1.58	2e−16	3e−15	30 x 30	

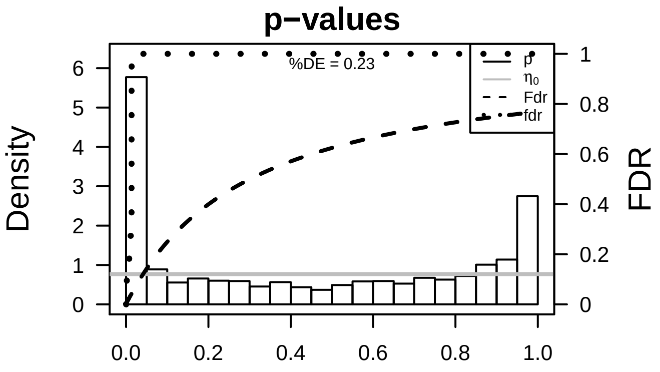

Supplement: Supplementary file 1 [file cells-09-00779-s001.zip › Supplementary materials/FileS4.zip!/FileS3/TFs - Results/Summary Sheets - Samples/Zhewan.1.25.1..pdf]
